# Transcriptomic insights into the shift of trophic strategies in mixotrophic dinoflagellate *Lepidodinium* in the warming ocean

**DOI:** 10.1093/ismeco/ycae087

**Published:** 2024-06-19

**Authors:** Jiawei Chen, Lixia Deng, Mengwen Pang, Yingdong Li, Zhimeng Xu, Xiaodong Zhang, Hongbin Liu

**Affiliations:** Department of Ocean Science, Hong Kong University of Science and Technology, Hong Kong SAR, China; Department of Ocean Science, Hong Kong University of Science and Technology, Hong Kong SAR, China; Department of Ocean Science, Hong Kong University of Science and Technology, Hong Kong SAR, China; Institute of Deep-sea Science and Engineering, Chinese Academy of Sciences, Sanya, China; Department of Ocean Science, Hong Kong University of Science and Technology, Hong Kong SAR, China; Department of Ocean Science, Hong Kong University of Science and Technology, Hong Kong SAR, China; Department of Ocean Science, Hong Kong University of Science and Technology, Hong Kong SAR, China; Hong Kong Branch of Southern Marine Science and Engineering Guangdong Laboratory (Guangzhou), Hong Kong SAR, China

**Keywords:** mixoplankton, dinoflagellate, transcriptome, temperature, phagocytosis

## Abstract

The shift between photoautotrophic and phagotrophic strategies in mixoplankton significantly impacts the planktonic food webs and biogeochemical cycling. Considering the projected global warming, studying how temperature impacts this shift is crucial. Here, we combined the transcriptome of in-lab cultures (mixotrophic dinoflagellate *Lepidodinium* sp.) and the metatranscriptome dataset of the global ocean to investigate the mechanisms underlying the shift of trophic strategies and its relationship with increasing temperatures. Our results showed that phagocytosis-related pathways, including focal adhesion, regulation of actin cytoskeleton, and oxidative phosphorylation, were significantly stimulated in *Lepidodinium* sp. when cryptophyte prey were added. We further compared the expression profiles of photosynthesis and phagocytosis genes in *Lepidodinium* sp. in the global sunlit ocean. Our results indicated that *Lepidodinium* sp. became more phagotrophic with increasing temperatures when the ambient chlorophyll concentration was >0.3 mg.m^−3^ (~20.58% of the ocean surface) but became more photoautotrophic with increasing temperatures when the chlorophyll concentration was between 0.2 and 0.3 mg.m^−3^ (~11.47% of the ocean surface). Overall, we emphasized the crucial role of phagocytosis in phago-mixotrophy and suggested that the expression profile of phagocytosis genes can be a molecular marker to target the phagotrophic activity of mixoplankton in situ.

## Introduction

Phago-mixotrophy (a combination of photoautotrophy and phagotrophy) is a common trophic strategy adopted by diverse marine unicellular plankton [1, 2]. Mixotrophic plankton (mixoplankton) can be grouped into two main functional categories: constitutive mixotroph (CM) and non-constitutive mixotroph (NCM) [1]. The former is described as phagotrophic phytoplankton that has inherited plastids and the capacity to ingest prey, whereas the latter is described as phototrophic zooplankton that acquires phototrophic capacity from its prey. Studies on mixotrophy over the past several decades stirred up our previous understanding of marine food webs based on the conventional view of species as either pure phototrophic or heterotrophic [3]. Recent modeling studies indicated that phago-mixotrophy profoundly impacts planktonic food webs and biogeochemical cycling, including the promotion of primary production, biomass transfer to higher trophic levels, and the functioning of the biological carbon pump [4–6].

Dinoflagellates are crucial members of marine plankton worldwide, distributing from cold arctic waters to warm tropical waters and from nutrient-replete coastal regions to nutrient-impoverished open oceans [7]. Phago-mixotrophy occurs among nearly all of the extent dinoflagellate orders [1, 8], and many mixotrophic dinoflagellates can form red tides, which leads to fish kills, oxygen depletion, and accumulation of toxins in the food chain [7]. It has been speculated that phago-mixotrophy is a crucial factor leading to the dominance of dinoflagellates in phytoplankton communities [9]. Since the late 1980s, great efforts have been paid to investigate the physiology of mixotrophic dinoflagellates, focusing on the environmental factors that stimulate ingestion and digestion under controlled conditions in lab experiments [2, 7, 8]. These studies are invaluable in helping the identification of mixoplankton species. However, a substantial lack of basic knowledge remains on the molecular mechanisms underlying phago-mixotrophy in dinoflagellates.

The relative contribution of photoautotrophic and phagotrophic processes in a single mixoplankton species can vary widely due to the selection between the two strategies under different abiotic and biotic conditions [2, 10]. Since phago-mixotrophy is a significant contributor to plankton dynamics, the flexible metabolisms of mixoplankton can profoundly impact global carbon sequestration [6, 11, 12]. Considering the projected global warming [13], understanding how temperature impacts the shift between trophic strategies is crucial. Wilken *et al.* (2013) [14] hypothesized that mixotrophic organisms become more heterotrophic with increasing temperatures. This hypothesis is derived from the metabolic theory of ecology (MTE), which predicts that heterotrophic metabolism is more temperature-sensitive than autotrophic metabolism [15, 16]. Although some studies using laboratory cultures have demonstrated that increasing temperatures lead mixoplankton to a more phagotrophic strategy, including chrysophyte *Ochromonas* sp. [14], dinoflagellate *Karlodinium veneficum* [17], dinoflagellate *Lepidodinium* sp. [18], and haptophyte *Isochrysis galbana* [19], some mixoplankton responded oppositely and become more photoautotrophic at higher temperatures, including dinoflagellate *Karlodinium armiger* and ciliate *Mesodinium rubrum* [20]. Additionally, MTE is based on a nutrient-sufficient condition, while nutrients are often limited in the open ocean. Therefore, whether the increase in temperature promotes the shift of mixoplankton to a more phagotrophic strategy in the natural ocean is still debatable.

To close these two knowledge gaps, we first conducted transcriptomic analysis on a CM dinoflagellate *Lepidodinium* sp., which has been demonstrated to become more phagotrophic at higher temperatures via in-lab experiments [18], to investigate the molecular mechanisms underlying the shift of trophic strategies. We further applied the expression profiles of photosynthesis and phagocytosis genes in *Lepidodinium* sp. to target photoautotrophic and phagotrophic activities, and used the global metatranscriptome dataset from *Tara* Oceans expedition to investigate whether the mixotrophic dinoflagellate *Lepidodinium* sp. become more phagotrophic with increasing temperatures in the global sunlit ocean.

## Materials and methods

### Algae cultures and microscopic observation

The mixotrophic dinoflagellate *Lepidodinium* sp. used in this study were isolated from Hong Kong coastal waters in April 2014. Dinoflagellate *Lepidodinium* sp. and cryptophyte prey *Rhodomonas salina* were cultured in 10% f/2 medium at 25°C, with a light intensity of 100 μmol photons.m^−2^.s^−1^ and a light:dark cycle of 12:12 h. *Lepidodinium* sp. cultures were maintained in two conditions, i.e., starved and well-fed conditions, to obtain cells under more photoautotrophic and more phagotrophic strategies, respectively. In the starved condition, no extra algal prey was provided, while in the well-fed condition, cryptophyte *R. salina* were added as prey every 12 h to maintain saturated prey density (~2000 cell.ml^−1^). Our previous study showed that inorganic nutrient concentration was negatively related to ingestion rate but positively related to the growth rate of *Lepidodinium* [18]. Therefore, we used 10 times diluted f/2 medium to balance the needs of active ingestion behavior (to capture gene expression signals) and high cell density (for nucleotide extraction).

*Lepidodinium* sp. cultures for RNA extraction were grown in eight 2 L polycarbonate bottles (Nalgene, MA, USA) (two for RNA-seq, three for the metatranscriptome of starved cultures, and three for the metatranscriptome of well-fed cultures), and cultures for physiological parameter measurements were grown in 12 75 cm^2^ cell culture flasks (SPL, Gyeonggi-do, Korea) (six for starved cultures and six for well-fed cultures). Semi-continuous cultures were transferred to new 10% f/2 media every 4 days when cell concentrations reached ~8000 cell.ml^−1^ (exponential phase). The exponential phase was determined based on the growth curve of *Lepidodinium*.

A subsample (1 ml) was collected from each culture flask every 24 h, fixed with Lugol’s solution (final concentration 2%), and observed under the microscope to determine cell density. The specific growth rate during exponential growth was calculated as [ln(N_1_)-ln(N_2_)]/(t_1_-t_2_), where N_1_ and N_2_ denote the abundances at time t_1_ and t_2_, respectively. To observe plastids and food vacuoles in cells, a ~ 2 ml live culture aliquot was filtered onto a 5-μm-pore black polycarbonate membrane (Whatman, Cytiva, Buckinghamshire, UK) by gravity. Membranes were placed on glass slides and observed under epifluorescence microscopy Olympus BX51 (Olympus, Tokyo, Japan) at 1000× magnification and 435 nm excitation.

### Photosynthesis and respiration

The effective quantum yield of PSII photochemistry (Y_II_) and electron transport rate (ETR) were determined using a Phytoplankton Pulse Amplitude Modified Fluorometer (Phyto-PAM-II, Walz GmbH, Effeltrich, Germany). Six replicates were measured for each treatment during the light period (well-fed cultures were measured after all prey was consumed), and each measurement was pre-acclimated in darkness for 5 min. Respiration rates were determined during the night period using a 24-channel SensorDish oxygen reader (PreSens, Regensburg, Germany). For each measurement (six replicates per treatment), 2 ml sensor vial (PreSens, Regensburg, Germany) was topped up with culture medium and closed tightly. The changes in oxygen concentration were monitored in each vial every 15 s for 6 h. Respiration rates were obtained from the slopes of the regression line of oxygen concentration versus time and then normalized to the cell abundance of each replicate. Shapiro–Wilk test was implemented using “Shapiro.test” function in R software [21] to test whether the data were normally distributed. We applied non-parametric tests using “Wilcox.test” to evaluate the differences among groups with abnormal distribution, and “t.test” to evaluate the differences among groups with normal distribution.

### RNA extraction, library construction, and sequencing

A total of 2 L culture samples (~8000 cell.ml^−1^) of each replicate was filtered onto a 10-μm-pore polycarbonate membrane (GVS, Roma, Italy) and transferred into 1 ml TRIzol reagent (Thermo Fisher Scientific, MA, USA) immediately. Total RNA was extracted using RNeasy Plus Kits (Qiagen, Hilden, Germany) according to the manufacturer’s protocol. RNA quality and integrity were tested using a NanoDrop spectrophotometer (Thermo Fisher Scientific, MA, USA), and an RNA Nano 6000 assay kit in conjunction with an Agilent Bioanalyzer 2100 system (Agilent Technologies, CA, USA), respectively. Only samples that met the requirements (260/280 ratio: 1.8–2.1, 260/230 ratio: 2.0–2.4, RIN value: >6.8) will be used further.

In-lab experimental design was showed in a conceptual diagram (See online supplementary material for a colour version of Fig. S1). In brief, to obtain a pure *Lepidodinium* sp. transcriptomic assembly, well-fed cultures were starved for two days to make sure all prey were consumed before sampling. Duplicate RNA samples were assigned to Poly(A) RNA sequencing (RNA-seq) in which only RNAs with poly(A) tails such as eukaryotic message RNAs will be selected, and poly-A oligo-attached magnetic beads were used for the library construction. Triplicate RNA samples from starved and well-fed cultures were assigned to metatranscriptome sequencing, in which all RNAs except ribosomal RNAs will be used for the library construction. RNA-seq and metatranscriptome sequencing were performed using a NovaSeq 6000 system (Illumina, CA, USA), and 150-bp paired-end reads were generated.

### Quality control, assembly, and annotation

Raw reads from RNA-seq duplicates were trimmed using Trimmomatic v0.39 [22] and then de-novo co-assembled using Trinity v2.14.0 [23]. Similar transcripts were clustered using CD-HIT [24] with “-c 0.95” parameter, and a single representative transcript was retained for each group. The quality of transcriptomic assembly was estimated using BUSCO v5.4.4 [25] with the lineage dataset “alveolata_odb10”. The longest open reading frame for each transcript was detected by TransDecoder v5.5.0 (https://github.com/TransDecoder/TransDecoder) and annotated against the Kyoto Encyclopedia of Genes and Genomes (KEGG) [26], TransportDB v2.0 [27], UniProt [28], Pfam A [29], and Clusters of Orthologous Genes [30] databases using Diamond v2.0.4 [31] with “--sensitive -e 1e-20” parameters. To distinguish proteins located in lysosomal membrane, proteins under Gene Ontology (GO) terms GO:0005765 (lysosomal membrane), GO:0061474 (phagolysosome membrane), and GO:0036020 (endolysosome membrane) with a qualifier tag “located in” were extracted based on QuickGO database [32]. Annotation results are listed in Table S1.

### Identification and global distribution

Internal transcribed spacer (ITS) and 18S sequences were extracted from the transcriptomic assembly using VSEARCH v2.22.1 [33] based on UNITE database v8.3 [34] and Protist Ribosomal Reference database (PR2) v5.0.0 [35], respectively. ITS sequences of *Lepidodinium* sp. and other known dinoflagellates were aligned using MUSCLE v3.8.31 [36] and trimmed using trimAl v1.2 [37]. The statistical selection of optimal phylogenetic models for nucleotides was implemented using jModelTest v.2.1.10 [38]. Maximum likelihood phylogenetic analysis was implemented using IQ-TREE v1.6.12 [39] with the ultrafast bootstrap parameter “-bb 1000” [40] and TIM + F + G4 model selected by jModelTest. Bayesian inference of phylogeny was performed using MRBAYES v3.2.7a [41].

Eukaryotic amplicon sequencing (18Sv9 rDNA) dataset from *Tara* Oceans expedition was downloaded from the European Bioinformatics Institute (EBI) under project PRJEB402. To evaluate *Lepidodinium* sp. and plankton abundance in global sunlit ocean, the amplicon reads from the euphotic layer (< 200 m) were aligned to the *Lepidodinium* sp. 18S sequence and PR2 database v5.0.0 using CoverM v0.6.1 (https://github.com/wwood/CoverM), respectively, under “contig” mode with parameters “--methods count --min-read-percent-identity 99 --min-read-aligned-percent 95”.

### Differential expression and enrichment analyses

Raw reads from metatranscriptomic triplicates were trimmed using Trimmomatic v0.39 [22] and then aligned to *Lepidodinium* sp. transcriptomic assembly using Bowtie2 v2.4.4 [42] with default settings. Based on the results (SAM files) from Bowtie2, the read counts of all genes at all samples were summarized using featureCounts v.2.0.0 [43] with the following parameters: “-M, -O, --fraction”. multidimensional scaling (MDS) analysis was implemented using the “plotMDS” function in R v4.1.2 [21]. To identify differentially expressed genes (DEGs) when *Lepidodinium* sp. shifts from more photoautotrophic (starved condition) to more phagotrophic (well-fed condition) strategy, a differential expression analysis was implemented using *edgeR* v.3.30.3 R package [44]. The TPM of all genes in the *Lepidodinium* sp. transcriptomic assembly is listed in Table S2. Only genes with an adjusted *P*-value < .01 and |log2 (foldchange)| > 1 were regarded as being DEGs. KEGG enrichment analysis was implemented using the “enricher” function in the *clusterProfiler* v.3.16.1 R package [45]. To identify the connection among pathways, k-means and hierarchical clustering analyses were conducted based on the involvement of genes using *factoextra* v1.0.7 [46] and *vegan* v2.6 [47] R packages, respectively. ANOSIM was implemented with the “anosim” function in R v4.1.2 [21].

### Global expression profiles and satellite observation

Metatranscriptome sequencing dataset for protists from *Tara* Oceans expedition was downloaded from the EBI under project PRJEB402. Metatranscriptome samples were trimmed using Trimmomatic v0.39 [22] and then aligned to *Lepidodinium* sp. transcriptomic assembly using Bowtie2 v2.4.4 [42] with default settings. Raw read counts were obtained using featureCounts v.2.0.0 [43]. *Tara* Oceans samples met the following acquirements were kept for further analysis: first, at least 0.1 million reads were aligned to *Lepidodinium* sp. transcriptomic assembly; second, samples were collected from the sea surface (depth of 5 m) to avoid significant variations in light intensity; third, the in-situ temperature of samples was ranged from 15°C to 31°C in which *Lepidodinium* sp. can grow. To compare the expression profiles among different samples, raw counts were converted to reads per kilobase million (RPKM) using *edgeR* R package v.3.30.3 [44]. Then, RPKM values were further converted to TPM as described previously [48]. To identify the correlations between co-expressed genes and environmental factors (traits), a weighted correlation network analysis (WGCNA) was implemented using the *WGCNA* v.1.71 R package [49] with a “signed” network type. Different soft thresholds were used in multi-WGCNAs, which were calculated using the “pickSoftThreshold” function based on a weighted correlation matrix (See online supplementary material for a colour version of Fig. S2). The 18-year sea surface chlorophyll observation data were collected by the MODIS sensor on the Aqua satellite of National Aeronautics and Space Administration (NASA), and the 0.5-degree data were downloaded from the earth observatory (https://earthobservatory.nasa.gov).

## Results and discussion

### Identification and distribution

**Figure 1:**
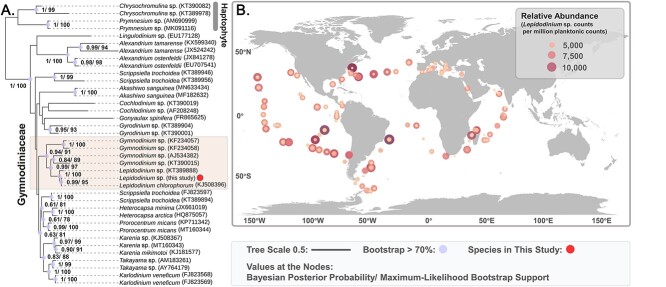
(**A**) Maximum likelihood phylogenetic tree based on the alignment of internal transcribed spacer (ITS) region among dinoflagellates. Sequences from haptophyte were set as out group. Accession numbers are provided for each sequence, and values at the nodes represent Bayesian posterior probability and maximum likelihood bootstrap support. (**B**) Global biogeography of *Lepidodinium sp.* based on the relative abundance of 18Sv9 rDNA sequences from the *Tara* oceans amplicon dataset.

The ITS sequence of *Lepidodinium* sp. was extracted from the transcriptome assembly in this study (110 879 genes detected, BUSCO completeness: 98.8% [S:26.3%, D:72.5%], F:0.6%, M:0.6%) and assigned for Maximum likelihood and Bayesian phylogenetic analyses. Our results showed that the species is a close relative to *Lepidodinium chlorophorum* (KJ508396) and belongs to the dinoflagellate family Gymnodiniaceae (Fig. 1A). Analysis of *Tara* Oceans amplicon (18Sv9 rDNA) dataset showed that *Lepidodinium* sp. was detected (contributing >0.1% planktonic 18S reads) in 90% of stations from euphotic layer (< 200 m) (Fig. 1B), indicating this species is widely distributed in the global ocean with ecological importance.

### Molecular mechanisms underlying physiological responses to the shift of trophic strategies

**Figure 2:**
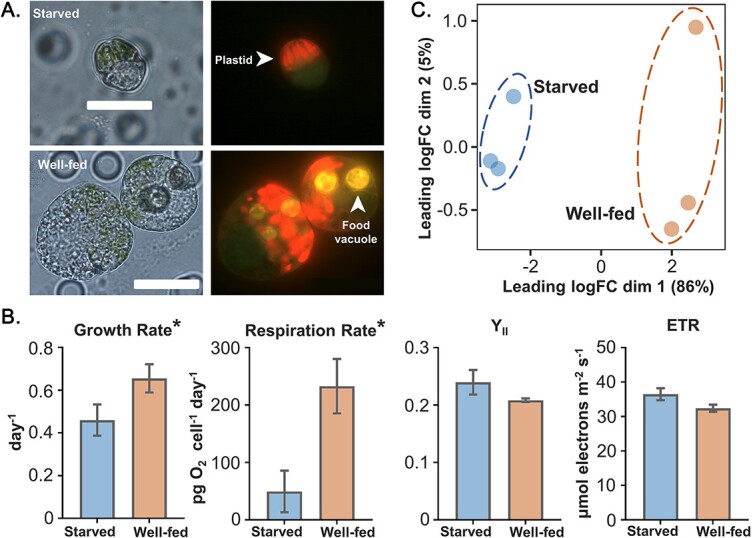
(**A**) Epifluorescence microscope images of starved and well-fed *Lepidodinium sp.* cells in bright field and 435 nm excitation under 1000× magnification with oil immersion (scale bar = 20 μm). Plastids and food vacuoles are marked by white arrows. (**B**) Growth rate, respiration rate, effective quantum yield of PSII photochemistry (Y_II_), and electron transport rate (ETR) of starved and well-fed samples. (**C**) Multidimensional scaling (MDS) plot based on the transcriptomic expression patterns of starved and well-fed samples. ^*^, *p*-value (between-group differences) < 0.05.

The mixoplankton *Lepidodinium* sp. cultures were maintained in two conditions, i.e. starved and well-fed conditions, to obtain cells under more photoautotrophic and more phagotrophic strategies, respectively. No extra algal prey was provided in the starved condition, while cryptophyte *R. salina* were added as prey in the well-fed condition. Starved and well-fed *Lepidodinium* sp. had obvious morphological and significant physiological differences. Through epifluorescence microscopy observation, food vacuoles can be observed clearly in all well-fed *Lepidodinium* sp. cells, and plastids can be observed in both starved and well-fed cells (Fig. 2A). The growth rate and respiration rate of well-fed cultures were significantly higher than starved cultures (*P*-value <.05, Fig. 2B). However, photosynthesis parameters, including the effective quantum yield of Y_II_ and ETR, did not show a significant difference between starved and well-fed *Lepidodinium* sp. (*P*-value >.05, Fig. 2B). Consistently, our previous study also indicated that the cellular chlorophyll *a* (Chl-*a*) content of *Lepidodinium* sp. grown in starved and well-fed conditions did not have significant differences [18].

To investigate if these physiological responses were supported by molecular evidence, we compared the gene expression between starved and well-fed *Lepidodinium* sp. cultures. The MDS plot showed that starved and well-fed samples were clustered into two separate groups (Fig. 2C). Additionally, the analysis of similarities (ANOSIM) also showed that the expression profiles were significantly different (*P*-value <.05) between the two conditions. A total of 1318 differentially expressed genes (DEGs) were detected, in which 1312 genes were significantly up-regulated and only six genes were significantly down-regulated in well-fed compared to starved samples (Table S3).

**Figure 3:**
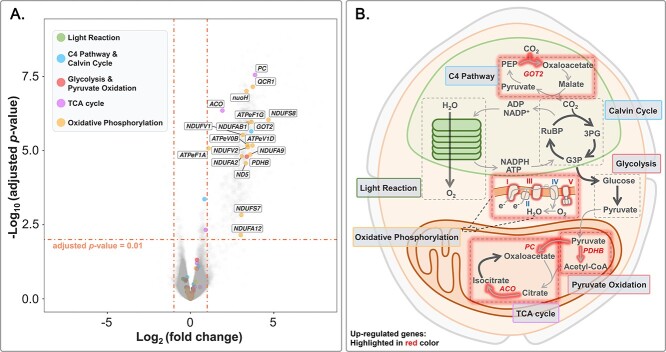
(**A**) Volcano plot of differentially expressed genes (DEGs) in well-fed compared to starved samples. DEGs involved in photosynthesis and cellular respiration are highlighted. (**B**) Schematic representation of the regulation of photosynthesis and cellular respiration processes in well-fed compared to starved samples.

First, we specifically focused on DEGs involved in the two strongly interconnected and high-profile processes, i.e. photosynthesis and cellular respiration [50]. Genes involved in light reaction, C4 pathway, and Calvin cycle were grouped in photosynthesis-related genes, whereas genes involved in glycolysis, pyruvate oxidation, tricarboxylic acid cycle, and oxidative phosphorylation were grouped in cellular-respiration-related genes. A total of 19 DEGs belonging to these processes were identified and highlighted in a volcano plot (Fig. 3A, Table S4). We did not find any DEGs involved in light reaction or Calvin cycle, and only one gene (*GOT2*) involved in C4 pathway was up-regulated (Fig. 3B). Among the other 18 genes, 15 of them were involved in oxidative phosphorylation, which consumes oxygen and produces adenosine triphosphate (ATP) [51]. The enrichment of DEGs in oxidative phosphorylation was consistent with the significantly higher respiration rate in well-fed than starved samples, while the insignificant regulation of photosynthesis-related genes was consistent with our photosynthetic parameter measurements.

### Biological processes stimulated by phagotrophy

**Figure 4:**
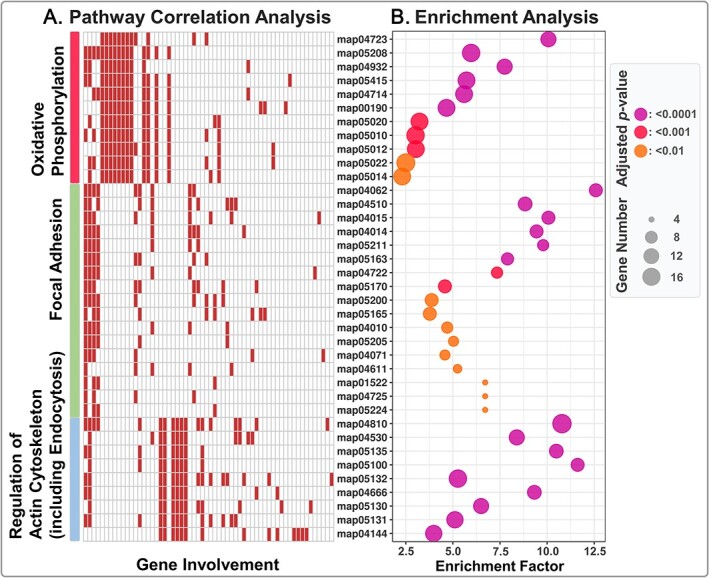
Results of enrichment and pathway correlation analyses. Map in the left panel indicates the involvement of genes in each pathway. Bubble plot in the right panel indicates the adjusted *p*-value, gene number, and enrichment factor of each KEGG pathway. KEGG, Kyoto Encyclopedia of genes and genomes.

**Figure 5:**
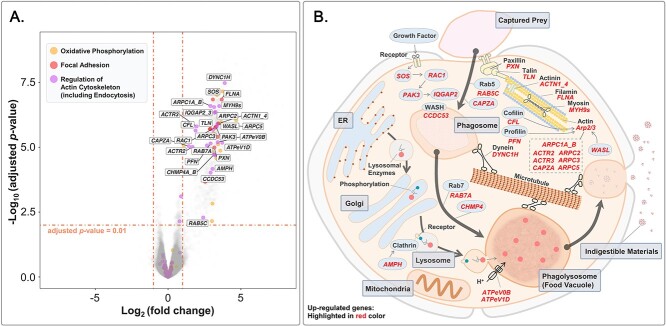
(**A**) Volcano plot of differentially expressed genes (DEGs) in well-fed compared to starved samples. DEGs involved in oxidative phosphorylation, focal adhesion, and regulation of actin cytoskeleton (including endocytosis) are highlighted. (**B**) Schematic representation of the focal adhesion and regulation of actin cytoskeleton (including endocytosis) processes in well-fed compared to starved samples.

We conducted KEGG enrichment analysis to identify key biological pathways stimulated by active ingestion. DEGs were significantly enriched (adjusted *P*-value <.01) in 37 pathways (Table S5). Since some pathways were enriched due to the same gene set, we conducted k-means and hierarchical clustering analyses based on the involvement of genes in each pathway to remove redundancy. Our results showed that the 37 KEGG pathways could be clustered into three main categories (Fig. 4): oxidative phosphorylation, focal adhesion, and regulation of actin cytoskeleton (including endocytosis). The clustering strategy was supported by ANOSIM with R = 0.99 and *P*-value <.001. DEGs involved in focal adhesion and regulation of actin cytoskeleton (including endocytosis) were labeled in a volcano plot (Fig. 5A, Table S6).

Focal adhesion provides a tight structural association between the actin cytoskeleton and the extracellular matrix [52]. Proteins involved in the construction of actin cytoskeleton, including paxillin (encoded by the gene *PXN*), talin (encoded by the gene *TLN*), actinin (encoded by the gene *ACTN1_4*), filamin (encoded by the gene *FLNA*), myosin (encoded by the gene *MYH9s*), and actin (encoded by the gene *ARP2/3*) [53, 54] were significantly up-regulated in well-fed samples (Fig. 5B). Moreover, the key regulator of cell–cell adhesion (IQ motif-containing GTPase-activating protein 2 [encoded by the gene *IQGAP2*]) [55] and its up-stream genes, including son of sevenless (*SOS*), ras-related C3 botulinum toxin substrate 1 (*RAC1*), and p21-activated kinase 3 (*PAK3*), were also highly expressed in well-fed cultures. Focal adhesion is an essential process in the capture of prey and engulfment. The tight intercellular physical interaction based on the actin cytoskeleton can facilitate the following phagocytosis.

Endocytosis is a widespread cellular process that regulates the uptake of exogenous molecules from extracellular regions [56]. Phagocytosis, a type of endocytosis, is used to describe the uptake of large particulates. In phagocytosis, prey is internalized by the invagination of the plasma membrane to form an intracellular vacuole known as the phagosome [57]. Genes involved in the formation of phagosome, including actin-related protein 2 (*ARP2/3*), WASH complex subunit (*CCDC53*) [58], capping actin protein of muscle Z-line alpha subunit 1(*CAPZA1*) [59], and ras-related protein Rab-5C (*RAB5C*) [60] were up-regulated in well-fed samples (Fig. 5B). Lysosomes are membrane-bound cell organelles that contain digestive enzymes. The gene amphiphysin (*AMPH*), which facilitates the formation of lysosomal clathrin coats, was also highly expressed in well-fed cultures [61]. Mature phagosomes become functional phagolysosomes, where prey is digested, by fusing with lysosomes. Genes involved in the maturity of phagolysosome, including ras-related protein Rab-7A (*RAB7A*) [62] and charged multivesicular body protein 4A/B (*CHMP4*) [63], were up-regulated in well-fed cultures. Moreover, we also detected the upregulation of lysosomal proton pumps V-type H^+^-transporting ATPase (encoded by the genes *ATPeV0B* and *ATPeV1D*) which acidify the lysosomal lumen to optimize the performance of digestive enzymes, and motor protein dynein (encoded by the gene *DYNC1H*) which functions in the transportation of phagolysosome [64]. Since focal adhesion and endocytosis are energy-demanding processes, the up-regulation of oxidative phosphorylation, which produces ATP, is reasonable and expectable.

The upregulation of genes involved in focal adhesion and regulation of actin cytoskeleton (including endocytosis), hereafter phagocytosis genes, can directly be linked to the activation of engulfment and digestion. Not only in CM dinoflagellate *Lepidodinium* sp., the upregulation of phagocytosis genes was also reported in NCM dinoflagellate *Dinophysis acuminata* [65] and heterotrophic dinoflagellate *Oxyrrhis marina* [66] when plenty prey was provided. Moreover, phagocytosis was also stimulated by feeding in cultured heterotrophic flagellate *Cafeteria burkhardae* [67] and in a natural heterotrophic flagellates community [68]. Considering phagocytosis is an ancient trait that marked the origin of eukaryotic cells [69], and many of the genes involved are evolutionarily conserved among protists [70, 71], the expression profile of phagocytosis genes can be regarded as a promising target to indicate relevant phagotrophic processes in natural mixoplankton communities [67]. Therefore, we used phagocytosis genes to target the phagotrophic activity of *Lepidodinium* sp. in the global ocean via the combination of metatranscriptome dataset from *Tara* Ocean expedition in our following analysis.

### The shift of trophic strategies with increasing temperatures in the global sunlit ocean

Wilken et al. (2013) [14] hypothesized that mixotrophic organisms become more heterotrophic with increasing temperatures. Its theory background is that metabolic processes are temperature dependent and can often be approximated by the Arrhenius equation, in which *Ea* is a quantitative measure of temperature dependence. Since the average *Ea* of heterotrophic metabolisms across a wide variety of different species (0.65 eV) is significantly higher than in terrestrial C3 plants (0.32 eV) [16], mixotrophic organisms were expected to display a stronger temperature response for heterotrophic than for autotrophic growth, and thus become more heterotrophic at higher temperatures. This hypothesis is debatable because some mixoplankton species did not follow this pattern [20], and its theoretical basis (MTE) was established on a nutrient-replete condition. Whether this hypothesis works in the natural ocean has yet to be proved. Our previous work has found that *Lepidodinium* sp. becomes more heterotrophic at higher temperatures via in-lab experiments [18]. Here, we aim to investigate if this shift can also be detected in situ via the combination of metatranscriptome dataset from *Tara* Oceans expedition and NASA’s satellite observations.

Apart from temperature, metabolic processes in mixoplankton can be impacted by other environmental factors, mainly including irradiance, prey abundance, Chl-*a*, and nitrate [10, 72, 73]. These variables need to be controlled for the investigation of temperature impacts. To minimize irradiance variance, only surface samples (5 m depth, collected during daytime) of *Tara* Oceans metatranscriptome dataset were selected for analysis [74]. A critical issue of our aim was to set criteria to differentiate prey abundance and nutrient concentration. Here, we choose Chl-*a* concentration as the criteria due to three reasons: first, nitrate concentration showed significant collinearity with Chl-*a* concentration (*P*-value <.001, See online supplementary material for a colour version of Fig. S3) among the selected *Tara* Ocean samples in this study; second, the abundance of algal prey can be reflected by Chl-*a* concentration directly; third, the sea surface Chl-*a* concentration can be observed by satellite, which facilitates potential modeling work in the future. Therefore, selected *Tara* Ocean samples were divided into four groups based on their Chl-*a* concentration: 0–0.1 (Chl-*a* ≤ 0.1), 0.1–0.2 (0.1 < Chl-*a* ≤ 0.2), 0.2–0.3 (0.2 < Chl-*a* ≤ 0.3), and > 0.3 mg.m^−3^. We set the interval as 0.1 mg.m^−3^ to make sure the sample size of each group was >20 to meet the requirement of weighted correlation network analysis (WGCNA), which was used to find clusters (modules) of highly correlated genes (genes in the same module have similar expression profile among all samples) and relate gene modules to environmental traits [49].

**Figure 6:**
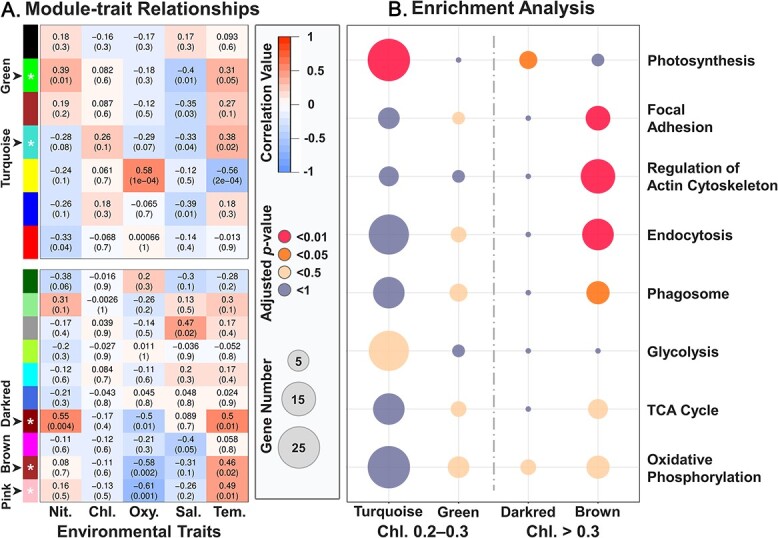
(**A**) Relationship between correlated gene modules and environmental traits when chlorophyll *a* (Chl-*a*) concentration is between 0.2–0.3 mg.m − ^3^ (upper figure) and higher than 0.3 mg.m − ^3^ (lower figure). The color of heatmap indicates the correlation value. Numbers in the brackets indicate the *p*-value of correlation. (**B**) KEGG enrichment analysis of gene modules significantly positively corelated to temperature. KEGG, Kyoto Encyclopedia of genes and genomes. Chl., Chl-*a* concentration.

Results of WGCNA showed that no gene module was significantly correlated to temperature when Chl-*a* concentration was <0.2 mg.m^−3^ (See online supplementary material for a colour version of Fig. S4). Two gene modules (green [61 genes] and turquoise [1834 genes]) and three gene modules (dark red [36 genes], brown [244 genes], and pink [94 genes]) were identified to have a significant positive correlation with temperature (*P*-value <.05) among samples within the Chl-*a* concentration range 0.2–0.3 and > 0.3 mg.m^−3^, respectively (Fig. 6A). We applied KEGG enrichment analysis to these gene modules and found that, when Chl-*a* concentration was between 0.2 and 0.3 mg.m^−3^, the photosynthesis pathway was significantly enriched (26 genes, adjusted *P*-value <.01) in module Turquoise but no pathway involved in phagocytosis was significantly enriched (Fig. 6B). However, when Chl-*a* concentration was >0.3 mg.m^−3^, pathways involved in phagocytosis, including focal adhesion (7 genes), regulation of actin cytoskeleton (16 genes), and endocytosis (13 genes), were significantly enriched (adjusted *P*-value <.01) in module brown (Fig. 6B). Although the photosynthesis pathway was detected in module dark red (adjusted *P*-value <.05) when Chl-*a* concentration was >0.3 mg.m^−3^, the number of detected photosynthesis genes (3 genes) in module dark red was much less than that (26 genes) in module turquoise.

**Figure 7:**
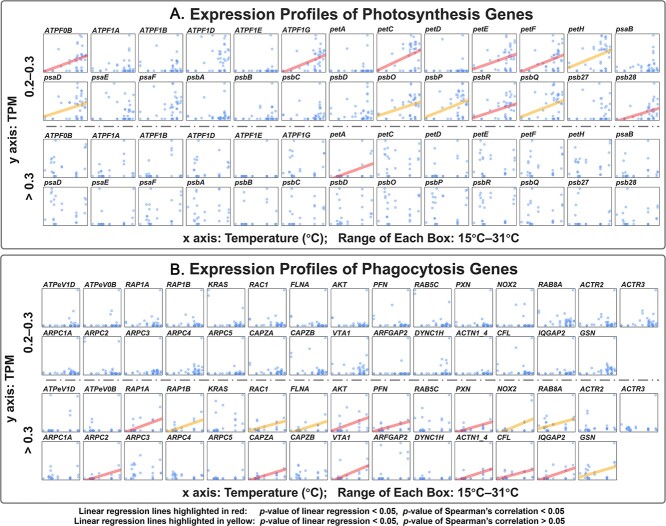
Expression profiles of photosynthesis (**A**) and phagocytosis (**B**) genes under two Chl-*a* concentration groups (0.2–0.3 and > 0.3 mg.M − ^3^). Each box indicates the relationship between temperature and transcripts per kilobase million (TPM) of one photosynthesis or phagocytosis gene.

To further validate WGCNA results, we extracted the photosynthesis (26 genes) and phagocytosis (29 genes) genes (Table S7) derived from the four modules and investigated their expression profiles along with increasing temperatures in different nutrient groups. Expression profiles of photosynthesis (Fig. 7A) and phagocytosis (Fig. 7B) genes under two Chl-*a* concentration groups (0.2–0.3 and > 0.3 mg.m^−3^) are shown via dot plot. Each box indicates the relationship between temperature (x axis, ranged from 15°C to 31°C) and expression level (transcripts per kilobase million [TPM]) (y axis) of one photosynthesis or phagocytosis gene. Linear regression lines are highlighted in red (the *P*-value of linear regression and Spearman’s correlation are <.05) or yellow (the *P*-value of linear regression is <.05 but Spearman’s correlation is >0.05) if significant positive relationship between temperature and TPM was detected. Our results showed that there was no significant positive correlation between gene expression and temperature when Chl-*a* concentration was <0.2 mg.m^−3^ (See online supplementary material for a colour version of Fig. S5), indicating that temperature may not be a key factor impacting the shift of trophic strategies when prey and nutrient were limited. When Chl-*a* concentration was between 0.2 and 0.3 mg.m^−3^, the expression of 12 photosynthesis genes significantly positively correlated to temperature (Fig. 7A), but no phagocytosis gene showed this pattern (Fig. 7B), suggesting that *Lepidodinium* sp. may increase reliance on photoautotrophic processes at higher temperatures. We supposed that the prey abundance was too low to support the cell growth in this condition, which reduces the favorability of grazing and phagotrophic investment. This finding was consistent with a recent modeling study which predicted that mixotrophs tend to evolve to become more reliant on phagotrophy at higher temperatures, but if prey abundance becomes too low, evolution favors greater reliance on photosynthesis [75]. When Chl-*a* concentration was >0.3 mg.m^−3^, only one photosynthesis gene significantly positively correlated to temperature (Fig. 7A), while the expression of 16 phagocytosis genes had significant positive correlations with temperature (Fig. 7B), suggesting that *Lepidodinium* sp. became more phagotrophic with increasing temperatures. Under this condition, the high Chl-*a* concentration indicates high nutrient concentration for photoautotrophy and high prey abundance for phagotrophy, which may fulfill the precondition of MTE and thus support Wilken’s hypothesis.

**Figure 8:**
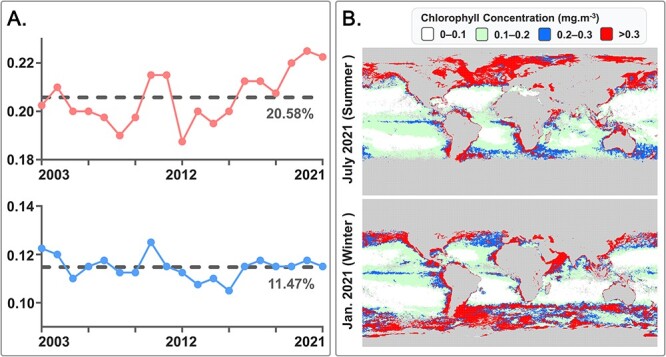
(**A**) The ratio of sea surface regions in which the chlorophyll *a* (Chl-*a*) concentration is higher than 0.3 mg.m − ^3^ (upper figure) and between 0.2–0.3 mg.m − ^3^ (lower figure) to the whole sea surface regions over the past 18 years. The dash lines indicate the mean value of 18-year observations from 2003 to 2021. (**B**) Color maps indicate the Chl-*a* concentration on the sea surface in July (summer) and January (winter) 2021.

In general, the results of WGCNA indicated that the correlation between temperature and the shift of trophic strategies in the natural sunlit ocean could be dependent on the prey abundance and nutrient concentration, which were indicated by Chl-*a* concentration in this study. We suggested that this may be the case for other mixotrophic dinoflagellates, and Chl-*a* concentration can be a useful criterion for Wilken’s hypothesis in the ocean. According to 18-year (2003–2021) observations of sea surface Chl-*a* concentration from NASA’s Aqua satellite, an average of 20.58% and 11.47% of sea surface’s Chl-*a* concentration were in the range of >0.3 and 0.2–0.3 mg.m^−3^, respectively (Fig. 8A). The global Chl-*a* concentration of two contrasted seasons are shown in Fig. 8B. Since the high Chl-*a* waters (> 0.3 mg.m^−3^) located mainly in the high latitude, it is likely that they will remain to be high in foreseeable future because of the reduced sea-ice extent and a longer phytoplankton growing season [76]. Assuming the areas of the ocean surface within the Chl-*a* concentration range > 0.3 and 0.2–0.3 mg.m^−3^ remain relatively constant, since the former (~20.58%) was nearly twice larger than the latter (~11.47%), a warming ocean may overall promote the shift of mixotrophic dinoflagellates to a more phagotrophic strategy and increase their emission of carbon dioxide.

## Conclusions

Phago-mixotrophy is a widespread ecological trait in marine protists. Investigating and modeling the planktonic food webs and biogeochemical dynamics involving mixoplankton are very challenging, mainly due to the flexible trophic strategies of mixoplankton. Although the physiology of mixoplankton can be investigated via in-lab experiments, we still need a more convenient approach to investigate phago-mixotrophy in global marine ecosystems in situ. One potential solution is to use the expression profile of marker genes that are indicative of phago-mixotrophic activity to target relevant processes of mixoplankton in the ocean. Through transcriptomic analysis of mixotrophic dinoflagellate *Lepidodinium* sp., we revealed that the pathways involved in phagocytosis, including focal adhesion, regulation of actin cytoskeleton (including endocytosis), and oxidative phosphorylation, were significantly enriched in well-fed samples compared to starved samples. Since phagocytosis is evolutionarily conserved and functionally relevant in other species, the expression profile of this process can be a promising marker for exploring phago-mixotrophic activity in mixoplankton in marine ecosystems.

By applying this marker in *Tara* Ocean metatranscriptome dataset, we tested the well-known hypothesis “mixotrophic organisms become more heterotrophic with increasing temperatures” in four regions based on their Chl-*a* concentration: 0–0.1, 0.1–0.2, 0.2–0.3, and > 0.3 mg.m^−3^. Our results showed that the expression of phagocytosis genes was only positively correlated to increasing temperatures when the ambient Chl-*a* concentration was >0.3 mg.m^−3^, while photosynthesis genes were positively correlated to increasing temperatures when the ambient Chl-*a* concentration was between 0.2 and 0.3 mg.m^−3^. These results suggested that the correlation between temperature and the shift of trophic strategies can be dependent on the prey abundance and nutrient concentration. Based on NASA's Aqua satellite's 18-year chlorophyll observations, an average of 20.58% and 11.47% of sea surface’s Chl-*a* concentration were in the range of >0.3 and 0.2–0.3 mg.m^−3^, respectively. Since the former region was nearly twice the size of the latter, the warming ocean may overall promote the shift of mixotrophic dinoflagellates to a more phagotrophic strategy and increase their carbon dioxide emission globally.

## Supplementary Material

Figure_S1_ycae087

Figure_S2_ycae087

Figure_S3_ycae087

Figure_S4_ycae087

Figure_S5_ycae087

Table_S1_ycae087

Table_S2_ycae087

Table_S3_ycae087

Table_S4_ycae087

Table_S5_ycae087

Table_S6_ycae087

Table_S7_ycae087

## Data Availability

The RNA-seq and metatranscriptome data of *Lepidodinium* sp. cultures have been deposited in National Centre for Biotechnology Information (NCBI) under the BioProject PRJNA940210. The ITS and 18S sequence of *Lepidodinium* sp. has been deposited in GenBank (accession: OQ534992 and PP669717, respectively). The transcriptomic assembly of *Lepidodinium* sp. has been deposited in National Omics Data Encyclopedia (NODE) under the project OEP003883. All bioinformatics commands and scripts are available via ‘https://github.com/jchenek/scripts-for-mixotrophic-dinoflagellate’. Authors declare that all data supporting the findings of this study are available within the article and its supplementary information files, or from the corresponding authors upon request.
