# Supplementary figures and images for: Transcriptomic insights into the shift of trophic strategies in mixotrophic dinoflagellate Lepidodinium in the warming ocean
Source: ISME Commun. 2024 Jun 19;4(1):ycae087. doi: 10.1093/ismeco/ycae087 (PMC11247192; doi:10.1093/ismeco/ycae087)

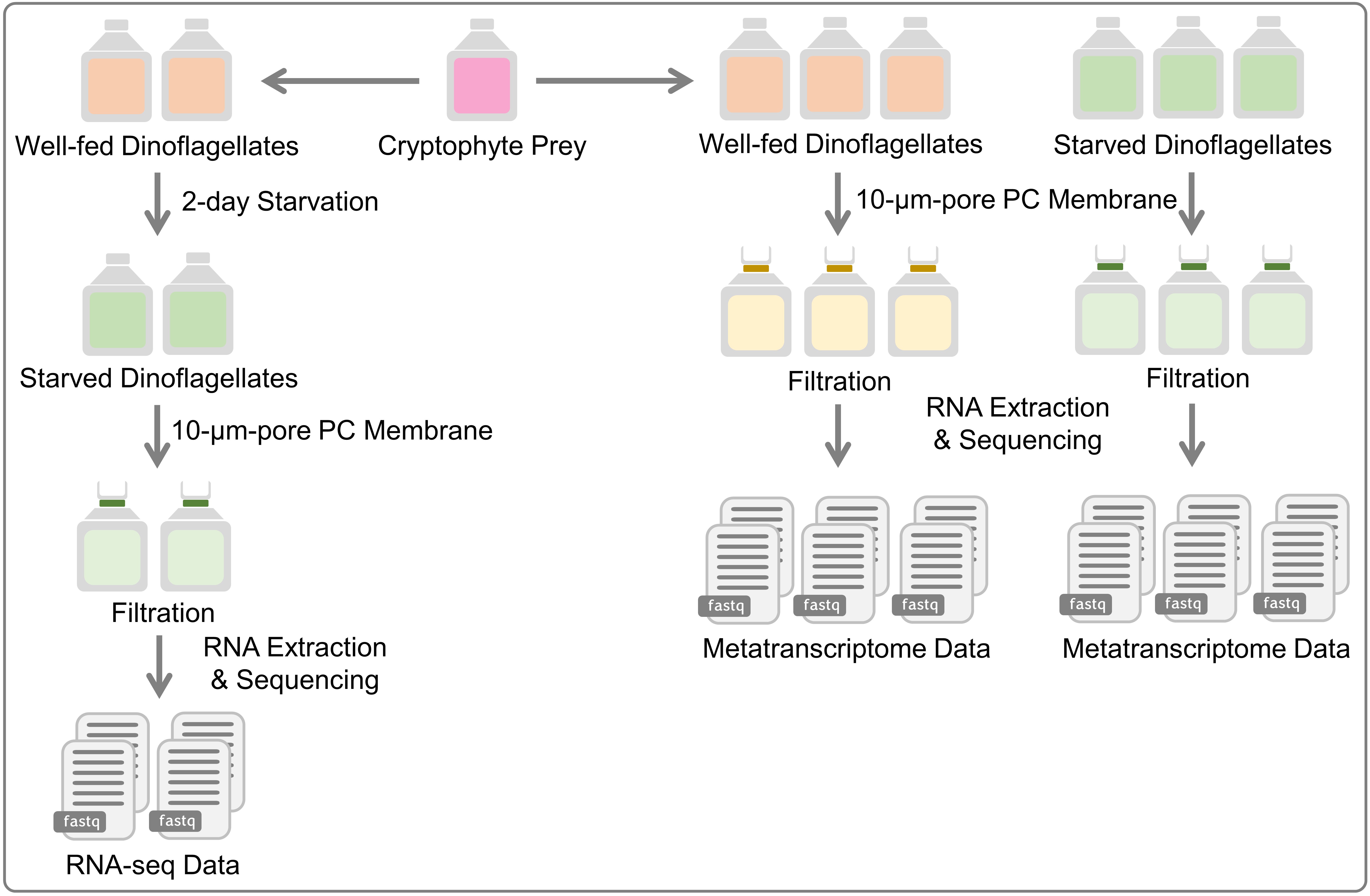

Supplement: Figure_S1_ycae087 [file figure_s1_ycae087.jpeg]

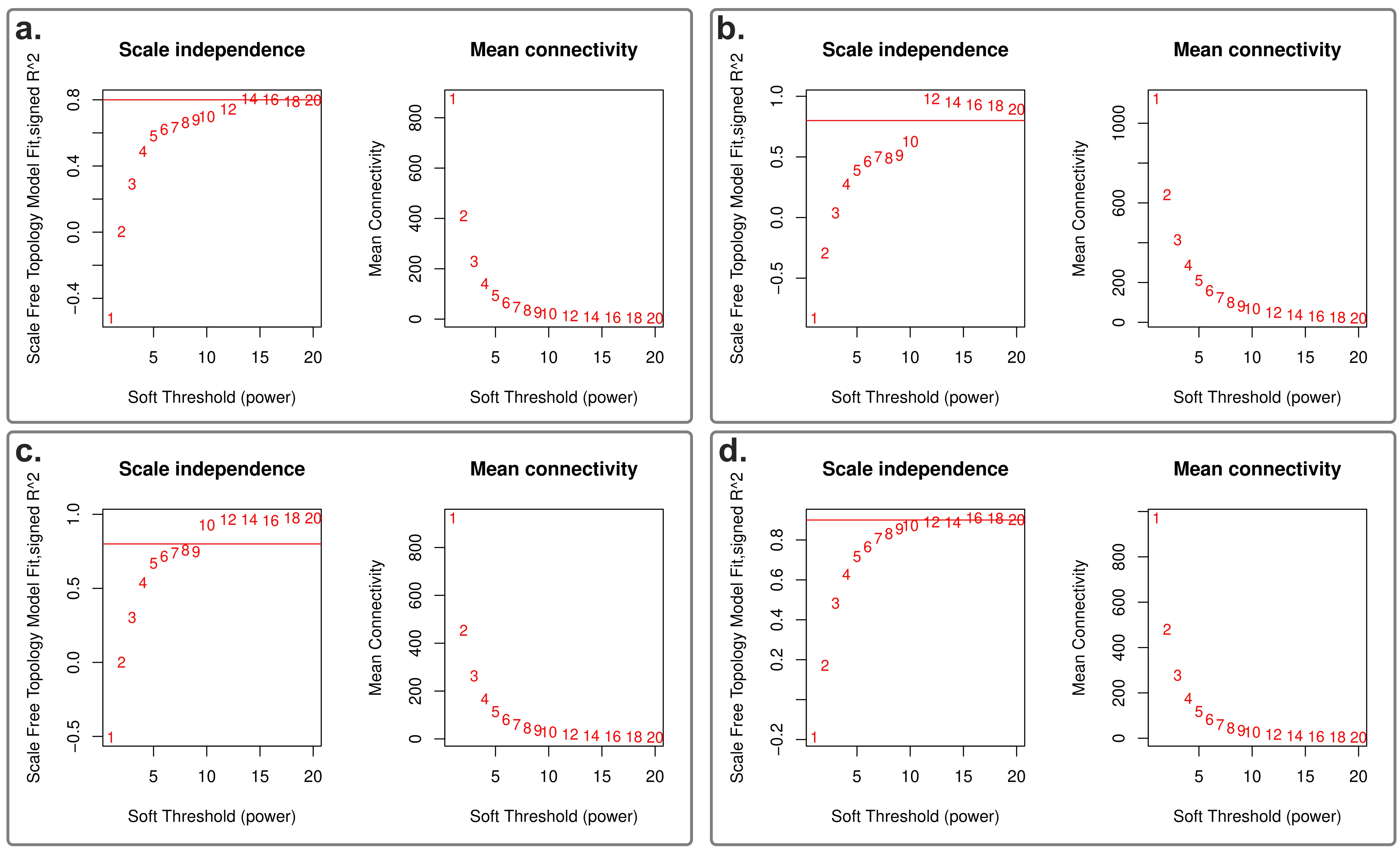

Supplement: Figure_S2_ycae087 [file figure_s2_ycae087.jpeg]

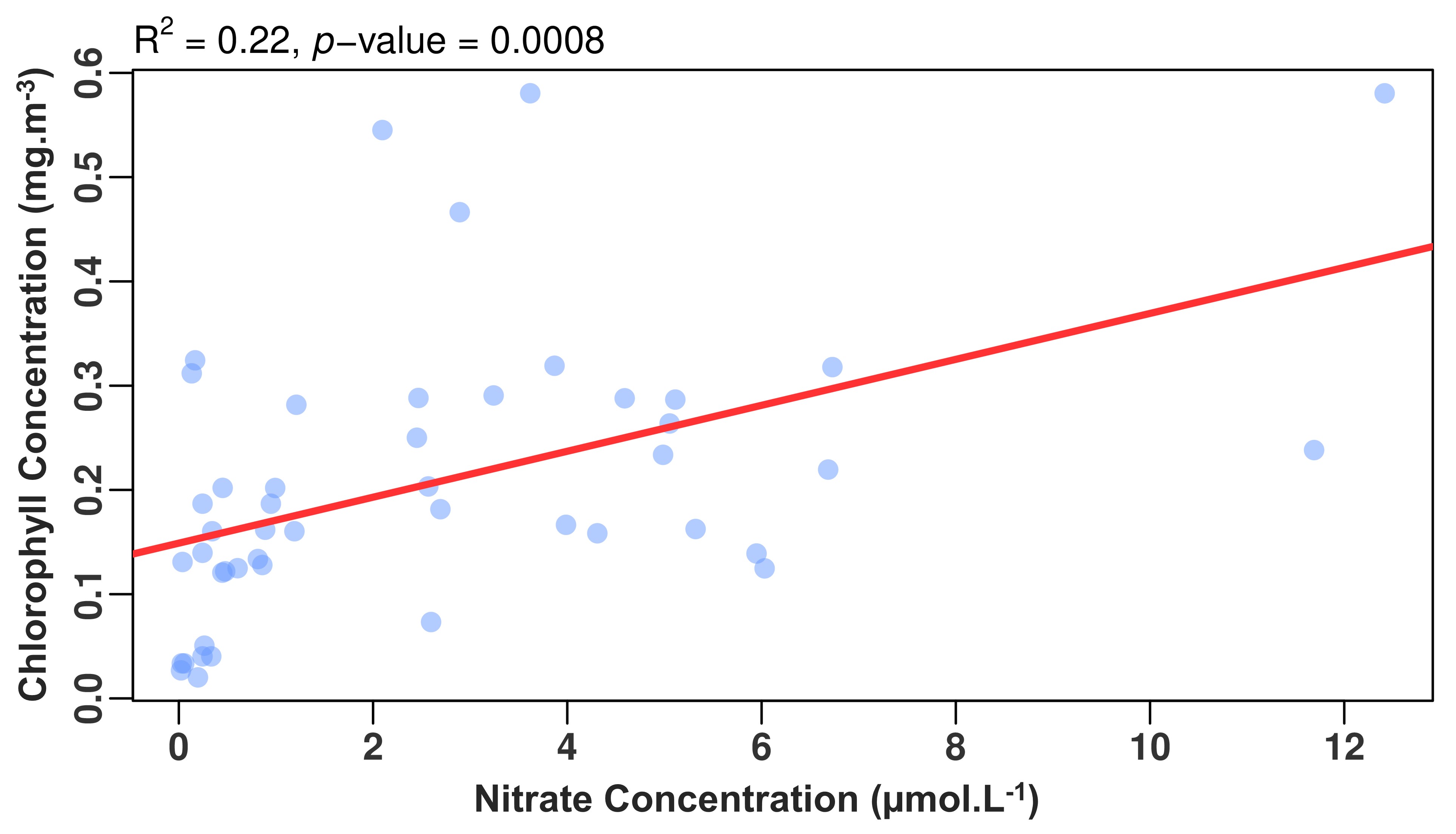

Supplement: Figure_S3_ycae087 [file figure_s3_ycae087.jpeg]

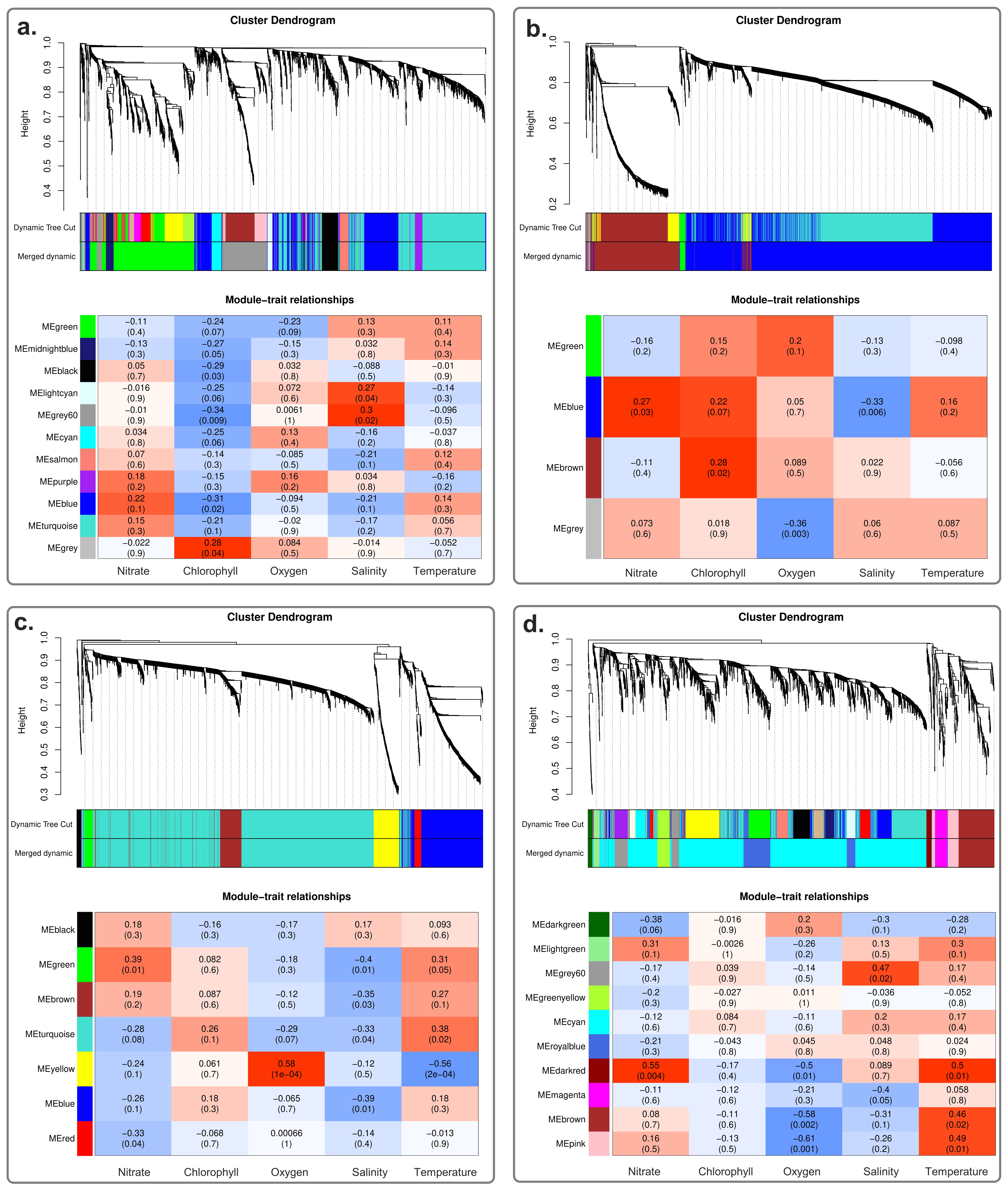

Supplement: Figure_S4_ycae087 [file figure_s4_ycae087.jpeg]

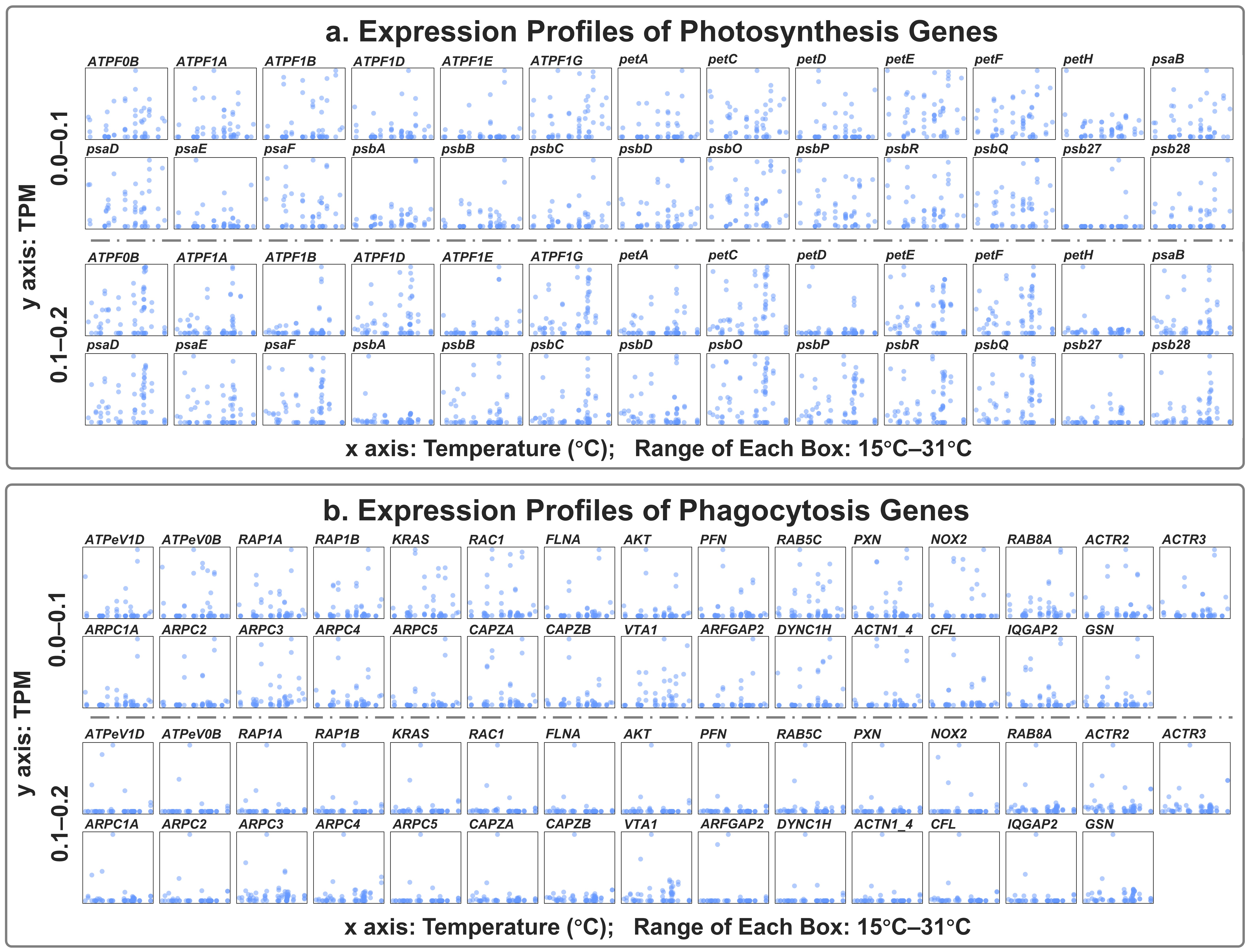

Supplement: Figure_S5_ycae087 [file figure_s5_ycae087.jpeg]
